# *Xiphovelopsis*, a new South American genus of Microveliinae (Hemiptera, Heteroptera, Gerridae), with the description of a new species

**DOI:** 10.1371/journal.pone.0313408

**Published:** 2024-11-18

**Authors:** Carla Fernanda Burguez Floriano, Juliana Mourão dos Santos Rodrigues, Felipe Ferraz Figueiredo Moreira

**Affiliations:** Laboratório de Entomologia, Instituto Oswaldo Cruz, Fundação Oswaldo Cruz, Rio de Janeiro, Brazil; Albany Museum, SOUTH AFRICA

## Abstract

Semiaquatic bugs of the subfamily Microveliinae (Hemiptera, Heteroptera, Gerridae) live in a wide range of habitats, including streams, rivers, lakes, lagoons, estuaries, mangroves, caves, crab holes, tree holes and bromeliads. A total of 120 species has been recorded from the Neotropical region, of which 11 bear modified pretarsal structures on the middle leg. They belong to the genera *Euvelia* Drake, 1957 (seven Neotropical species), *Husseyella* Herring, 1955 (three Neotropical species), and *Xiphovelia* Lundblad, 1933 (mainly Asian genus with one Neotropical species assigned to it). Here, we describe *Xiphovelopsis* Floriano & Moreira, **gen. nov.** to include *Xiphovelopsis tarumana* Floriano & Moreira, **sp. nov.** and *Xiphovelopsis lacunana* (Drake & Plaumann, 1953) **comb. nov.**, which is removed from the genus *Xiphovelia* due to the morphological differences and the geographic isolation in relation to its current congeners. We also provide images and notes on *Euvelia* and *Husseyella*.

## Introduction

Semiaquatic bugs of the subfamily Microveliinae (Hemiptera, Heteroptera, Gerridae) live in a wide range of habitats, including streams, rivers, lakes, lagoons, estuaries, mangroves, caves, crab holes, tree holes and bromeliads [1, 2]. The taxon was included in Veliidae until Armisén *et al*. [3] transferred it to Gerridae, with which it shares the reduced number of tarsomeres and the presence of a fecundation pump on the female gynatrial complex [1].

A total of 120 species has been recorded from the Neotropical region, distributed in the genera *Aegilipsovelia* Polhemus, 1970 (three species); *Euvelia* Drake, 1957 (seven species); *Microvelia* Westwood, 1834 (106 species); *Husseyella* Herring, 1955 (three species); and *Xiphovelia* Lundblad, 1933 (one species). *Aegilipsovelia* is restricted to northern and western Mexico [4], *Euvelia* is esclusively South American [5, 6], *Microvelia* is cosmopolitan [1], *Husseyella* is found along the coasts of southern Florida to southern Brazil [7, 8], and *Xiphovelia* is mainly Asian with one South American species assigned to it [9–12].

Current knowledge on the habits of *Xiphovelia* is scarce. The Asian species have been collected on pool areas of streams and rivers, while *X*. *lacunana* (Drake & Plaumann, 1953) was collected on rivers of the Paraná and Uruguay basins, southwestern Brazil [13, 14]. This species can be distinguished from other South American Microveliinae based on the pretarsal claws and ventral arolium of the middle leg modified into three blade-like structures [10]. Individuals of *Euvelia* and *Husseyella* have the claws and ventral arolium modified into four blade-like structures (the ventral arolium is split into two blades), while in *Microvelia* the claws are sickle-shaped and the arolia are similar to setae, which is the plesiomorphic condition [1].

Although modified pretarsal claws and arolia are notable structures, they probably evolved independently in several other lineages of Microveliinae, such as *Mangrovelia* Linnavuori, 1977, from western Africa; *Pseudovelia* Hoberlandt, 1950, from the oriental region; and *Xiphoveloidea* Hoberlandt, 1950, from continental Africa and Madagascar [1, 15]. Andersen [1] noted, however, that the split ventral arolium is restricted to *Euvelia* and *Husseyella*, which could indicate a single origin.

This fact was corroborated by Armisén *et al*. [3], who recovered the two as sister-groups and this clade sister to *Microvelia*. The monophyly of *Euvelia* was challenged, however, because the three described species of *Husseyella* were nested within it in a clade sister to *E*. *discala* Polhemus & Polhemus, 1984. The morphological differences between these two genera are subtle and they might eventually prove to be synonyms, with *Husseyella* representing just a halophilous lineage within the freshwater *Euvelia*. Because Armisén *et al*. [3] only included American Microveliinae belonging to three genera in their analysis, the phylogenetic relationships within this subfamily remain largely unresolved.

*Xiphovelia lacunana* was originally described in *Microvelia*, then transferred to *Xiphovelia* by Polhemus [14]. Andersen [1] was the first author to question this generic placement and others expressed the same doubt later, including Zettel [16] and Ye & Bu [11, 12]. However, while studies on Asian *Xiphovelia* are advancing, the knowledge on *X*. *lacunana* is stagnant.

Here, we describe *Xiphovelopsis* Floriano & Moreira, **gen. nov.** to include *Xiphovelopsis tarumana* Floriano & Moreira, **sp. nov.** and *Xiphovelopsis lacunana* **comb. nov.**, which is removed from the genus *Xiphovelia* due to the morphological differences and the geographic isolation in relation to its current congeners. We also provide images and notes on *Euvelia* and *Husseyella*.

## Material and methods

This study is based on dry specimens deposited in the following institutions: Coleção Entomológica do Instituto Oswaldo Cruz, Fundação Oswaldo Cruz, Rio de Janeiro, Brazil (CEIOC); Coleção de Invertebrados, Instituto Nacional de Pesquisas da Amazônia, Manaus, Brazil (INPA); Museu Paraense Emílio Goeldi, Belém, Brazil (MPEG); Museu de Zoologia, Universidade de São Paulo, São Paulo, Brasil (MZUSP); and National Museum of Natural History, Smithsonian Institution, Washington D.C. United States (NMNH). In the NMNH, series of photographs were taken using a Cannon EOS 5D and combined into multi-focal images using the Visionary Digital Software. In the same institution, micrographs were obtained using a Zeiss EVO/MA15 scanning electron microscope (SEM). In this case, specimens were glued to standard SEM stubs, sputter coated, and examined. Distribution maps were created using Qgis 2.6.1. All images were edited using Adobe Photoshop and Adobe Illustrator CS6.

Abbreviations used are as follows: body length (BL), head length (HL), head width through the eyes (HW), length of antennomeres I–IV [excluding interarticular pieces] (ANT I, ANT II, ANT III, ANT IV), eye width (EYE), pronotal length (PL), pronotal width (PW), femoral length (FEM), tibial length (TIB), tarsomere length (TAR I, TAR II). All measurements are given in millimeters and were always taken in their maximum dimensions.

## Results and discussion

### *Xiphovelopsis* Floriano & Moreira, gen. nov.

(Figs 1–6).

**Fig 1:**
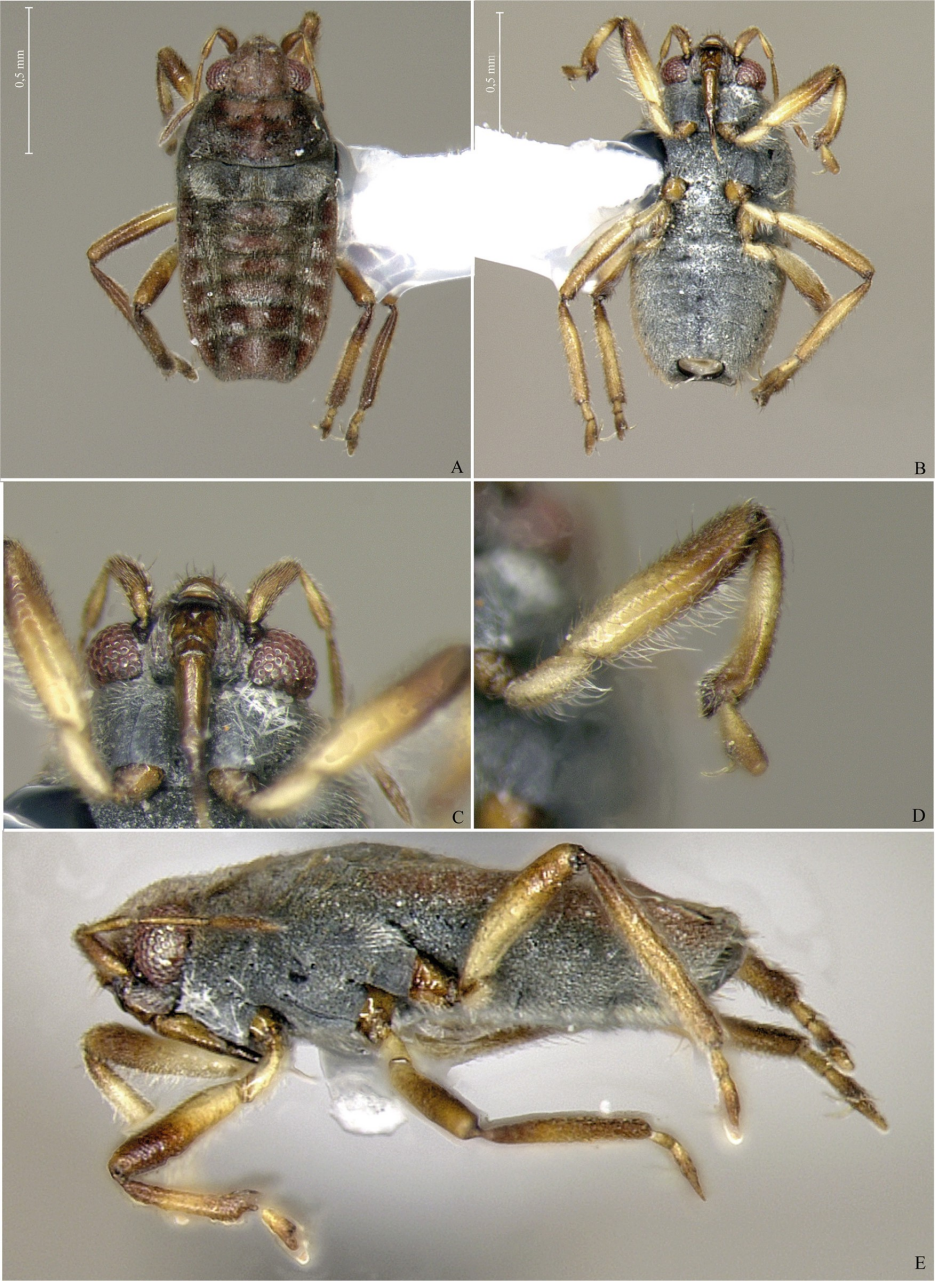
*Xiphovelopsis tarumana* sp. nov., holotype male. **A.** Habitus, dorsal view. **B.** Habitus, ventral view. **C.** Habitus, lateral view. **D.** Anterior leg, lateral view. **E.** Grooming comb.

**Fig 2:**
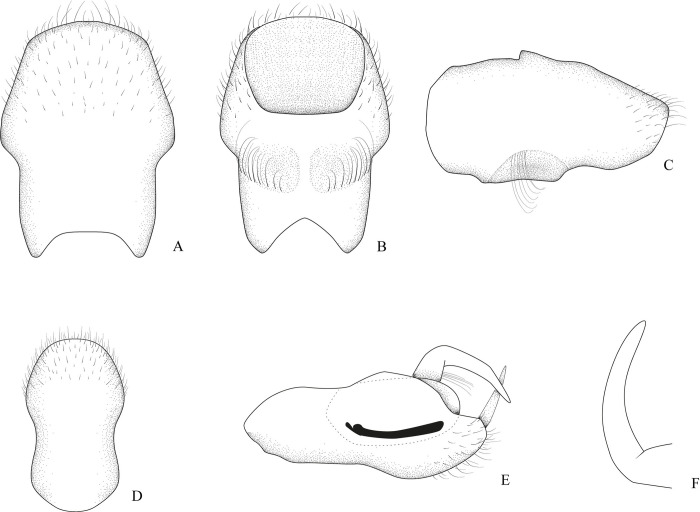
*Xiphovelopsis tarumana* sp. nov.. holotype male. **A.** Abdominal segment VIII, dorsal view. **B.** Abdominal segment VIII, ventral view. **C.** Abdominal segment VIII, lateral view. **D.** Proctiger, dorsal view. **E.** Pygophore, lateral view. **F.** Paramere.

**Fig 3:**
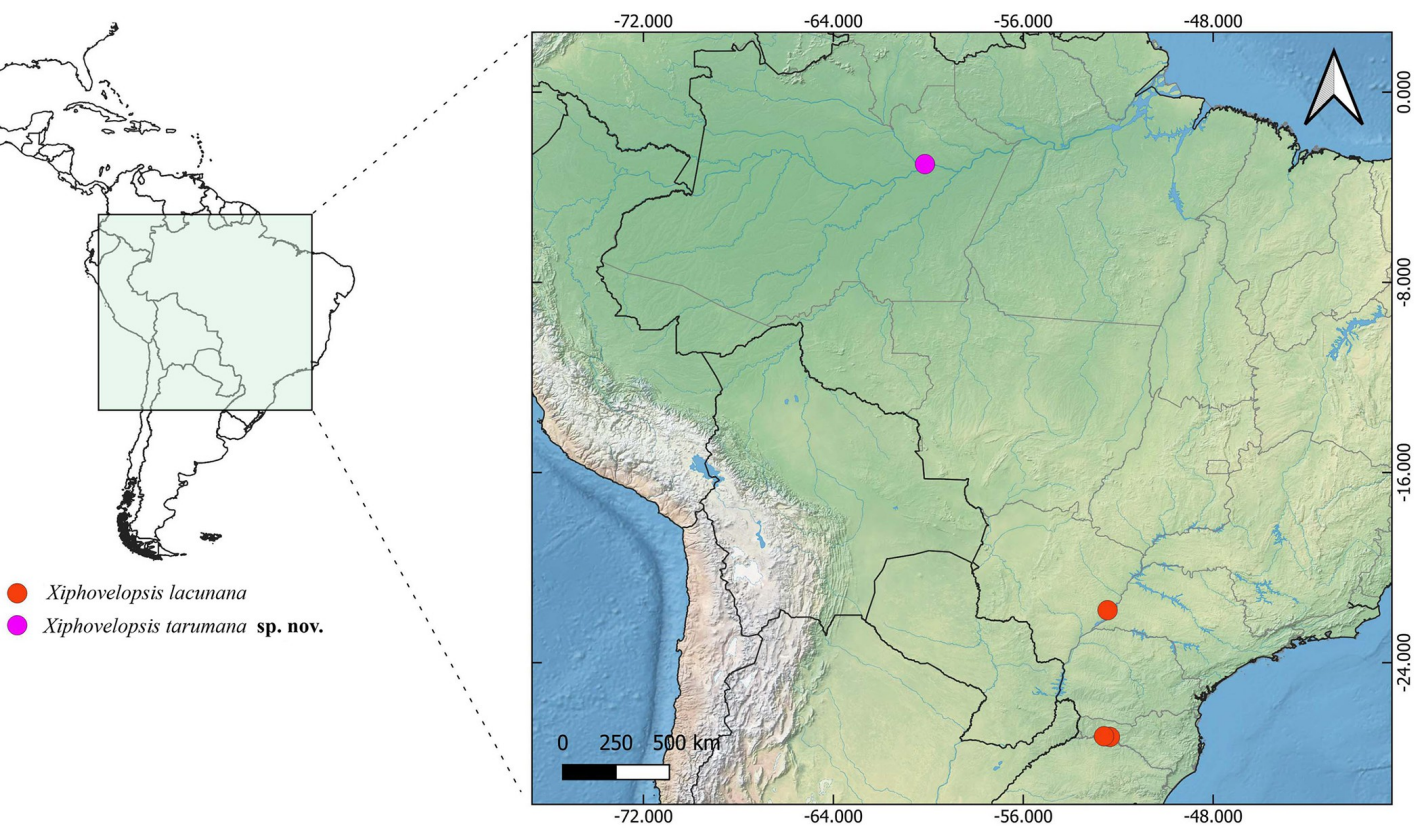
Geographic distribution of *Xiphovelopsis* gen. nov.. Spatial data from Natural Earth (http://www.naturalearthdata.com/).

**Fig 4:**
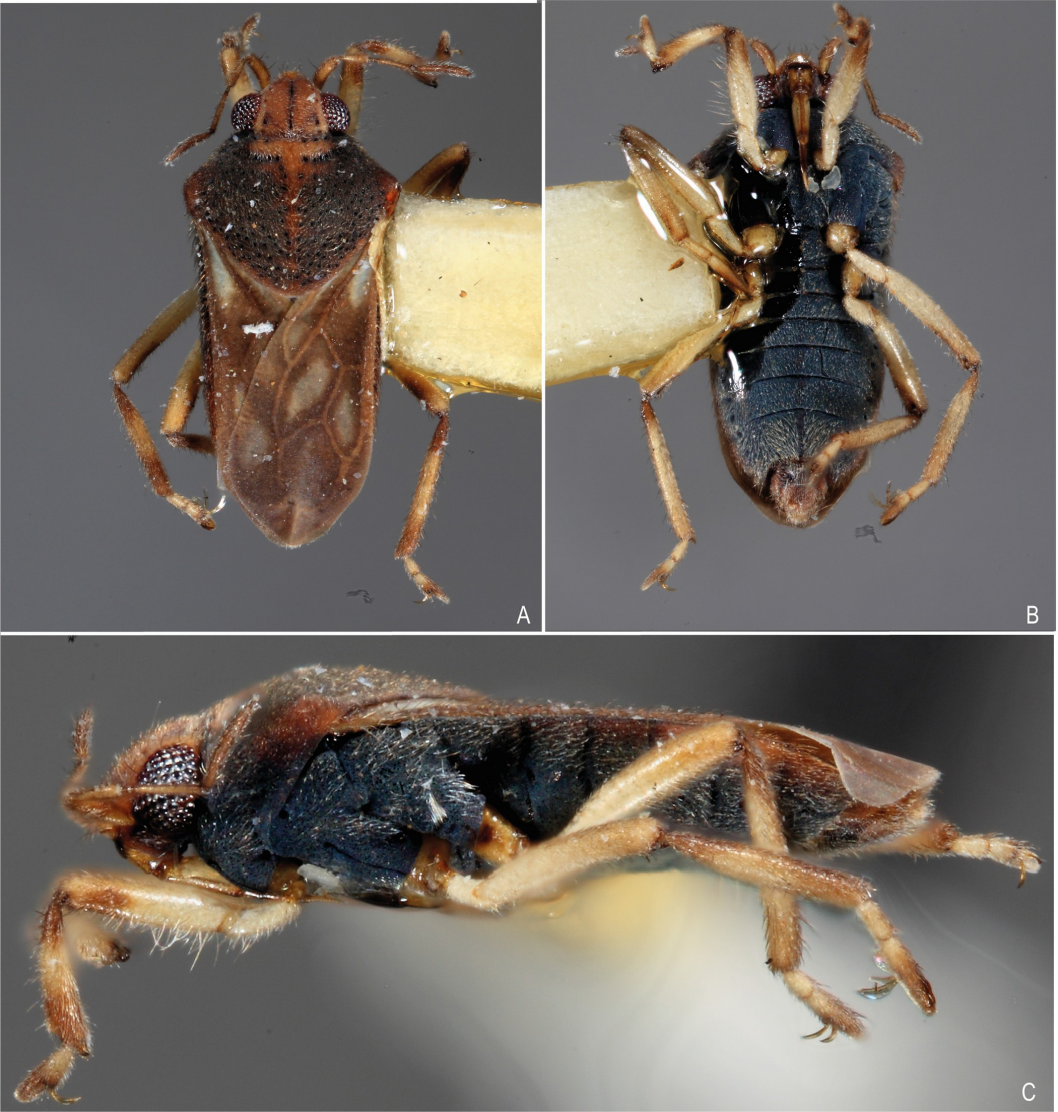
*Xiphovelopsis lacunana* comb. nov., holotype male, habitus. **A.** Dorsal view. **B.** Ventral view. **C.** Lateral view.

**Fig 5:**
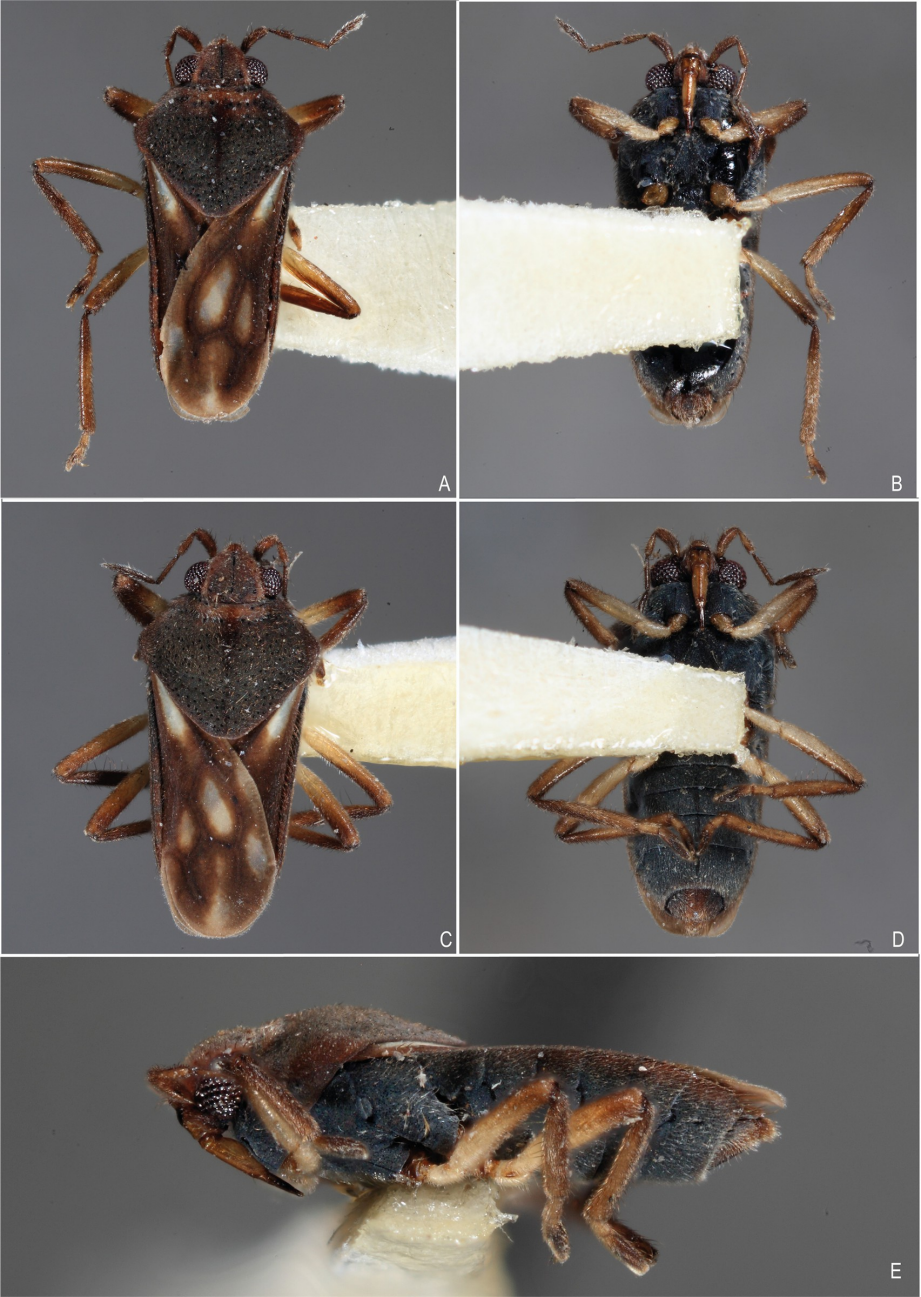
*Xiphovelopsis lacunana* comb. nov., habitus. **A.** Male, dorsal view. **B**. Male, ventral view. **C.** Female, dorsal view. **D.** Female, ventral view. **E.** Female, lateral view.

**Fig 6:**
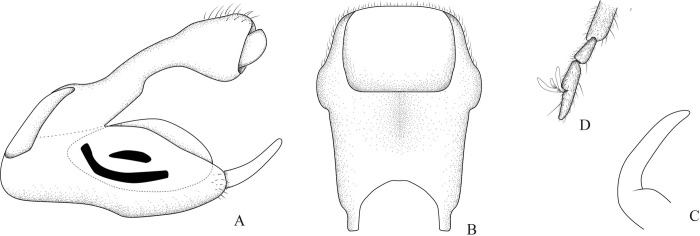
*Xiphovelopsis lacunana* comb. nov. **A.** Male, terminalia, lateral view. **B.** Male, abdominal segment VIII, ventral view. **C.** Male, paramere, lateral view. **D.** Middle tarsus.

#### Diagnosis

Body length 2.09–2.40. Forewing with four closed cells. Fore femur with long setae ventrally; setae longer than femur width. Male fore tibia with short grasping comb and apical spur. Middle femur with 90–100% hind femur length. Middle tibia with 70–80% hind tibia length. Middle tarsomere I 1.2–1.4 times longer than hind tarsomere I. Middle tarsomere II 1.6–1.9 times longer than middle tarsomere I. Middle pretarsal claws blade-like; middle ventral arolium modified into one blade-like structure. Male paramere long, subequal in length to pygophore height, slightly curved; lateral margins converging; apex acute.

#### Description

Body small, oval (Figs 1, 4 and 5); length 2.09–2.40. Body length/width ratio 2.40–2.90. General color brown to black; legs yellow to brown.

Head declined anteriorly, with three pairs of trichobothria inserted into deep pits and short setae dorsally, with longer setae anteriorly; median sulcus well defined (Figs 1A, 4A, 5A and 5C). Eye globose; maximum width half of minimum interocular width; without ocular setae. Labium reaching middle of mesosternum; article I almost three times longer than II; III twice as long as I and IV. Antennal length 0.85–0.91; antennomere I thickened, slightly curved laterally, with thick setae on mesal margin; II thicker on distal 3/4; III thinner than others, with parallel lateral margins; I and III subequal in length; IV fusiform, longer than others, 1.88–2.13 times longer than II.

Thoracic pleura and sterna covered by punctures and scattered silvery setae, without black denticles, tubercles or projections. Metasternal scent gland opening (omphalium) not visible; scent channels prominent, curving slightly anterad to base of metacetabula; evaporatory area with tuft of setae. Trochanters with long setae ventrally; some setae subequal in length to trochanter width (Figs 1D, 4B and 5D). Fore femur longer than fore tibia, with long setae on posterior surface; some setae longer than femur width. Fore tibia slightly curved, with a distal depression; male grasping comb short but projected beyond tibial apex (Figs 1B, 1D and 4B); male grooming comb at distal depression (Fig 1D). Middle femur thinner than fore and hind femora, longer than middle tibia, subequal in length to hind femur. Middle tibia with 70–80% hind tibia length. Middle tarsomere I 1.2–1.4 times longer than hind tarsomere I. Middle tarsomere II 1.6–1.9 times longer than middle tarsomere I. Middle pretarsal claws blade-like; middle ventral arolium modified into one blade-like structure (Figs 4B, 4C and 6D); claws subequal in length to tarsomere I. Hind femur shorter than hind tibia. Hind tibia 1.2–1.3 times longer than middle tibia. Hind tarsomere II 1.7–2.3 times longer than hind tarsomere I. Middle and hind legs, except trochanters, covered by setae distinctly shorter than those on fore femur; tibiae also with long, thin setae centrally; these as long as those on fore femur.

Lateral margins of abdomen converging posteriorly. Abdominal sides and venter covered by silvery setae; sterna V–VII with glabrous midline. Posterior margin of male abdominal segment VII rounded, unmodified. Male terminalia symmetrical (Figs 2 and 6); posterodorsal margin of abdominal segment VIII rounded (Fig 2A); venter of segment VIII with one or two depressions (Fig 2B and 2C). Male proctiger without basolateral processes; posterior margin rounded (Fig 2D). Male pygophore rounded on posterior margin. Male paramere long, subequal in length to pygophore height, slightly curved; lateral margins converging; apex acute (Figs 2E, 2F, 6A and 6C). Male phallus with long ventral sclerite (Fig 6A).

#### Macropterous form

Pronotum with a yellowish to orange transverse stripe anteriorly, sometimes extending along midline of posterior lobe; humerus and posterior margin orange-brown or light brown. Forewing brown, with a whitish macula within each cell, one adjacent to posterior margin, and one on the distal portion (Figs 4 and 5).

Pronotum subpentagonal, elevated medially, with deep punctures throughout surface (punctures as in Fig 8E and 8F); humerus slightly elevated; posterior margin rounded; anterior portion with sparse silvery setae (Figs 4A, 5A and 5C). Wings surpassing apex of abdomen, with four closed cells (Figs 4A, 5A and 5C).

Abdomen dorsally covered by wings, except laterotergites II–VI.

#### Apterous form

Orange-brown stripe on anterior lobe of pronotum thicker than on macropterous form, extending posteromedially.

Pronotum subtrapezoid, extended posteriorly, completely covering meso- and metanota; posterior margin slightly curved (Fig 1A); short setae and silvery pubescence laterally and posteriorly; punctures adjacent to anterior margin, a row on anterior 1/3, scattered over posterior 2/3.

Abdominal mediotergite I longer than II–VI, with posterior margin slightly concave medially, covered by silvery setae laterally and silvery pubescence centrally; II, III, V, and VI subequal in length, each between 0.14–0.15; IV shorter, 0.12 in length; VII longer, 0.25 in length; sides of II–VII with silvery setae and pubescence; II–IV each with a pair of silvery pubescent areas submedially; V–VII each with single silvery pubescent area medially (distinctly wider on V). Abdominal laterotergites III–VI with silvery setae posteriorly.

#### Type species

*Microvelia lacunana* Drake & Plaumann, 1953, by present designation.

#### Geographic distribution

Brazil, states of Amazonas, Mato Grosso do Sul, and Santa Catarina (Fig 3).

#### Etymology

The generic name refers to its resemblance to the genus *Xiphovelia*.

#### Comments

*Microvelia lacunana* was originally described from southeastern state of Mato Grosso do Sul [13], then had its distribution expanded south to western state of Santa Catarina [14]. In this same study, it was transferred to *Xiphovelia* based on the flattened body and the head and eyes closely appressed to the pronotum. The author noted, however, that it differed from Asian representatives of the genus by the lack of apterous specimens and the saber-like male paramere.

Andersen [1] questioned this transference and cited the species as *Microvelia lacunana* throughout his monograph. According to him, the male of this species lacked the earlike lateral projections of the pygophore found in all *Xiphovelia* males. He also noted a proximity between *M*. *lacunana*, *Euvelia*, and *Husseyella*. However, the claws and split ventral arolium are modified into four blade-like structures in the middle pretarsus of the last two genera, while *M*. *lacunana* displays only three blade-like structures because the ventral arolium is not split. He concluded that *M*. *lacunana* probably deserved an exclusive taxonomic positioning separated from *Euvelia*, *Husseyella*, and *Xiphovelia*. Later, Ye & Bu [11] revised Asian *Xiphovelia*, described three species, and questioned the permanence of *X*. *lacunana* in this genus, stating that the only shared character would be the flattened body. Such doubt was also expressed by Zettel [16].

After examining the holotype of *M*. *lacunana*, and considering the morphological differences and the geographic isolation in relation to its current congeners, we propose erecting *Xiphovelopsis* Floriano & Moreira, **gen. nov.** It can be distinguished from *Xiphovelia* based on the following features: 1) body length 2.09–2.40 in *Xiphovelopsis* (vs. 1.50–2.30); 2) pronotum completely covering the meso- and metanota (Fig 1A) (vs. not covering); 3) male grasping comb short (Fig 1D) (vs. long, reaching up to 1/3 of tibial length); 4) middle leg longer than hind leg (vs. shorter); 5) fore femur with long setae subequal to its width (vs. without such setae); 6) male pygophore lacking earlike lateral projections (vs. with earlike lateral projections); and 7) male paramere long, curved, subequal in length to pygophore height (Figs 2E and 6C) (vs. paramere short, rudimentary). Based on the pretarsal modifications, the new genus might be allied to *Husseyella* and *Euvelia*, as suggested by Andersen [1], but this remains to be tested in a phylogenetic context.

### *Xiphovelopsis tarumana* Floriano & Moreira, sp. nov.

(Figs 1–3).

#### Diagnosis

Body length 2.40; body color mainly brown; venter of male abdominal segment VIII with a pair of depressions and a row of setae.

#### Type material examined

Holotype. BRAZIL–**Amazonas** • Manaus, Tarumã-Mirim, Rio Negro; [−02.97, −60.20]; 03-05.X.2002; D.L.V. Pereira leg.; apterous ♂, INPA.

#### Description

BL 2.40 (terminalia removed); HL 0.35; HW 0.52; ANT I 0.20; ANT II 0.17; ANT III 0.22; ANT IV 0.32; EYE 0.11; PL 0.37; PW 0.75; fore leg: FEM 0.50, TIB 0.42, TAR I 0.20; middle leg: FEM 0.55, TIB 0.50, TAR I 0.12, TAR II 0.20; hind leg: FEM 0.60, TIB 0.65, TAR I 0.10, TAR II 0.17.

Head and antenna brown. Eye reddish. Clypeus and labial articles I–III light brown; labrum and labial article IV blackish. Pronotum dark brown; thick orange-brown stripe on anterior portion extending posteromedially (Fig 1A). Thoracic pleura, sterna and acetabula black. Coxae dorsally brown. Coxae ventrally, trochanters, and basal half of femora yellowish. Tibiae brown, lighter centrally. Fore tarsus yellowish on basal half; middle and hind tarsomere I yellowish; middle and hind tarsomere II brown, yellowish basally. Abdominal tergum I black; II black, brown on center of mediotergite; III–IV blackish laterally; rest of medio- and laterotergites brown. Abdominal segment VIII and proctiger dorsally yellow, blackish posteriorly. Abdominal sides and venter black. Ventrally exposed terminalia brown.

Head covered by short setae, with silvery pubescence centrally and long setae anteriorly; length subequal to labial article I; impressed midline and trichobothrial insertions evident. Antenna covered by short setae; antennomere I slightly curved laterally, thicker than others; II thicker on apex; III narrower than others; IV fusiform. Labium glabrous, reaching middle of mesosternum.

Pronotum extended posteriorly, completely covering meso- and metanota, with short setae and silvery pubescence laterally and posteriorly; punctures adjacent to anterior margin, a row on anterior 1/3, scattered over posterior 2/3; posterior margin slightly curved (Fig 1A). Fore acetabulum anteriorly and hind acetabulum posteriorly with silvery setae. Thoracic sterna with silvery setae, without elaborate ornamentation, black spinules, or punctures. Coxae with short, barely perceptible setae; rest of legs covered by short setae; trochanters ventrally and fore femur with long setae almost as long as respective segment width (Fig 1D). Fore femur thicker than middle femur, narrower than hind femur. Fore tibia slightly curved, with distal depression; male grasping comb short but projected beyond tibial apex (Fig 1B and 1D); grooming comb at distal depression (Fig 1D). Hind femur not surpassing posterior abdominal margin, with black spinules ventrally. Tarsi cylindrical; middle and hind tarsomere I shorter than respective tarsomere II; middle pretarsal claws blade-like; middle ventral arolium modified into one blade-like structure.

Abdominal mediotergites unpunctured; I with silvery setae laterally and silvery pubescence centrally; sides of II–VII with silvery setae and pubescence; sides of II–VII with silvery setae and pubescence; II–IV each with a pair of silvery pubescent areas submedially; V–VII each with single silvery pubescent area medially (distinctly wider on V). Abdominal laterotergites III–VI with silvery setae posteriorly; III–V dull, VI and VII shiny; posterior angle of last laterotergite not acutely projected posteriorly (= without connexival spine). Abdominal sides and venter unpunctured; sterna VI and VII slightly depressed centrally. Posterior margin of male abdominal segment VII rounded, unmodified. Male terminalia symmetrical (Fig 2); posterodorsal margin of abdominal segment VIII rounded (Fig 2A); venter of segment VIII with two depressions and a row of setae (Fig 2B and 2C). Male proctiger without basolateral processes; posterior margin rounded (Fig 2D). Male pygophore rounded on posterior margin. Male paramere long, subequal in length to pygophore height, slightly curved; lateral margins converging; apex acute (Fig 2E and 2F).

#### Etymology

The specific epithet is derived from the type locality.

#### Geographic distribution

Brazil, state of Amazonas (Fig 3).

#### Comments

This new species can be distinguished from *X*. *lacunana* based on the following features: 1) lighter general color (Fig 1 vs. Figs 4 and 5); 2) lack of golden setae on abdominal sternum VII (Fig 1B) (vs. with golden setae; Fig 4B); and 3) venter of male abdominal segment VIII with a pair of depressions and a row of setae (Fig 2B and 2C) (vs. with one depression, without row of setae; Fig 6B).

### *Xiphovelopsis lacunana* (Drake & Plaumann, 1953)

(Figs 3–6).

*Microvelia lacunana* Drake & Plaumann, 1953 [13]: 415–416.

*Xiphovelia lacunana*; Polhemus [14]: 646–647.

*Xiphovelopsis lacunana* **comb. nov.**

#### Diagnosis

Body length 2.09; body color mainly black; abdominal sternum VII covered by golden setae; venter of male abdominal segment VIII with one depression, without row of setae.

#### Type material examined

Holotype. BRAZIL–**Mato Grosso** [**do Sul**] • [Bataguassu], [Rio Caraguatá]; [−21.80, −52.45]; [F. Plaumann leg.]; macropterous ♂, NMNH.

#### Additional material examined

BRAZIL–**Santa Catarina** • [Seara], Nova Teutônia; [−27.13, −52.35]; VI-2-1957; F. Plaumann leg.; 1 macropterous ♂, 1 macropterous ♀, NMNH.

#### Measurements

BL 2.09; HL 0.30; HW 0.55; ANT I 0.19; ANT II 0.15; ANT III 0.19; ANT IV 0.32; EYE 0.13; PL 0.70; PW 0.98; fore leg: FEM 0.51, TIB 0.40, TAR I 0.19; middle leg: FEM 0.62, TIB 0.55, TAR I 0.13, TAR II 0.25; hind leg: FEM 0.62, TIB 0.68, TAR I 0.09, TAR II 0.21.

#### Geographic distribution

Brazil, states of Mato Grosso do Sul and Santa Catarina.

### *Euvelia* Drake, 1957

**Fig 7:**
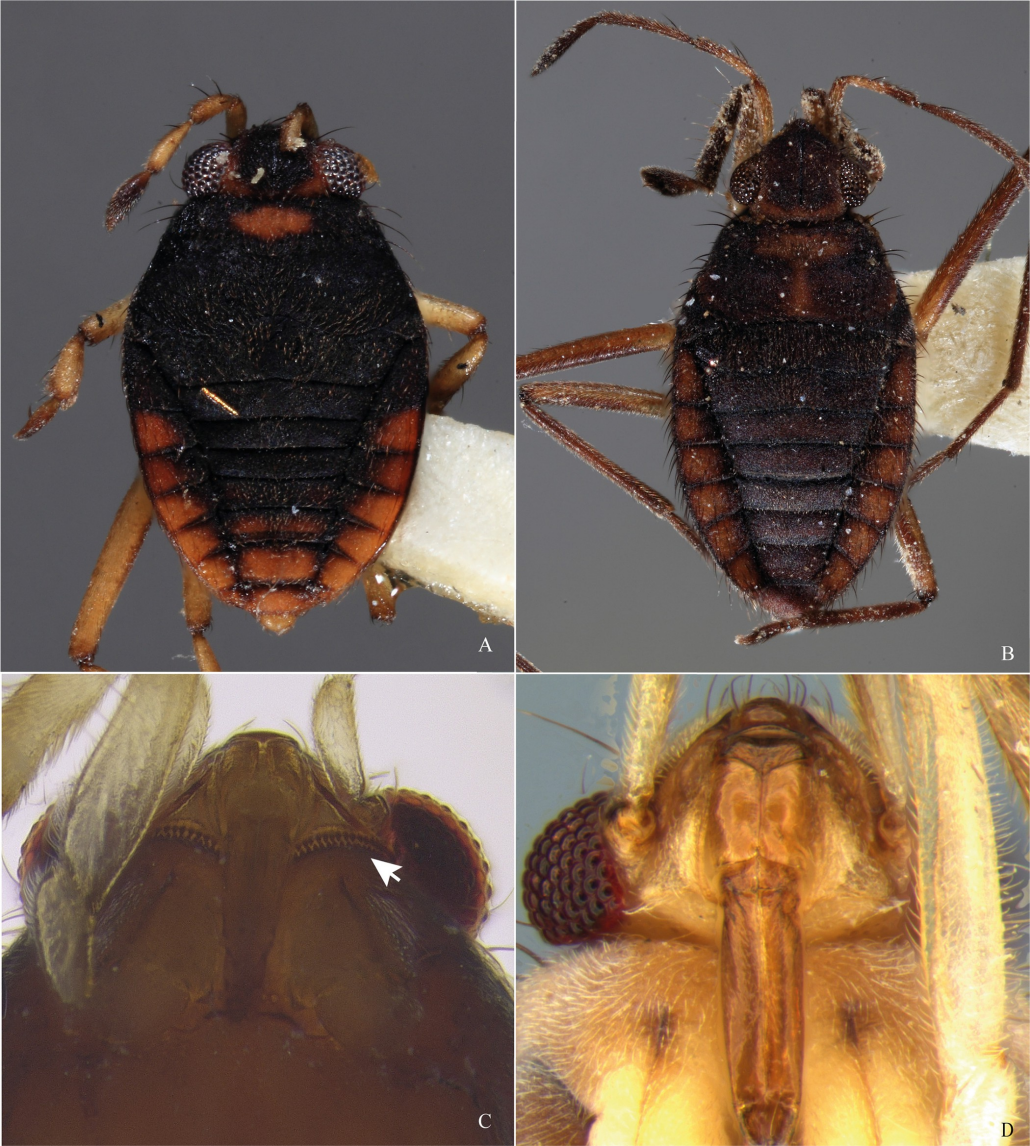
Microveliinae. **A, C.** *Euvelia advena*. **B, D.** *Husseyella diffidens*. **A**–**B.** Habitus, dorsal view. **C**–**D.** Head and thoracic sterna, ventral view.

**Fig 8:**
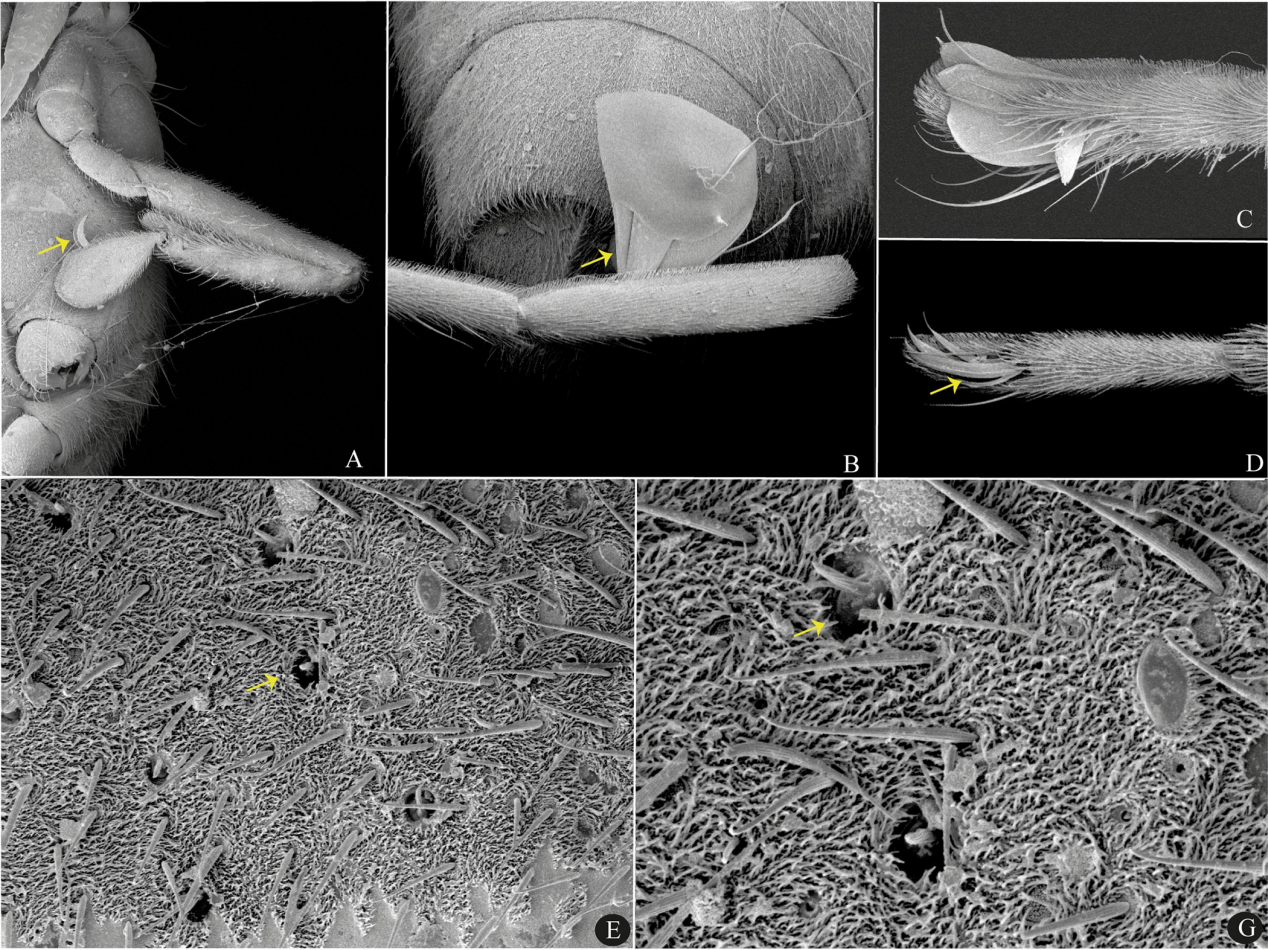
*Euvelia mazzuconiae*, female, scanning electron micrographs. **A.** Anterior leg, ventral view. **B.** Middle tarsomere II. **C**–**D**. Pronotum.

**Fig 9:**
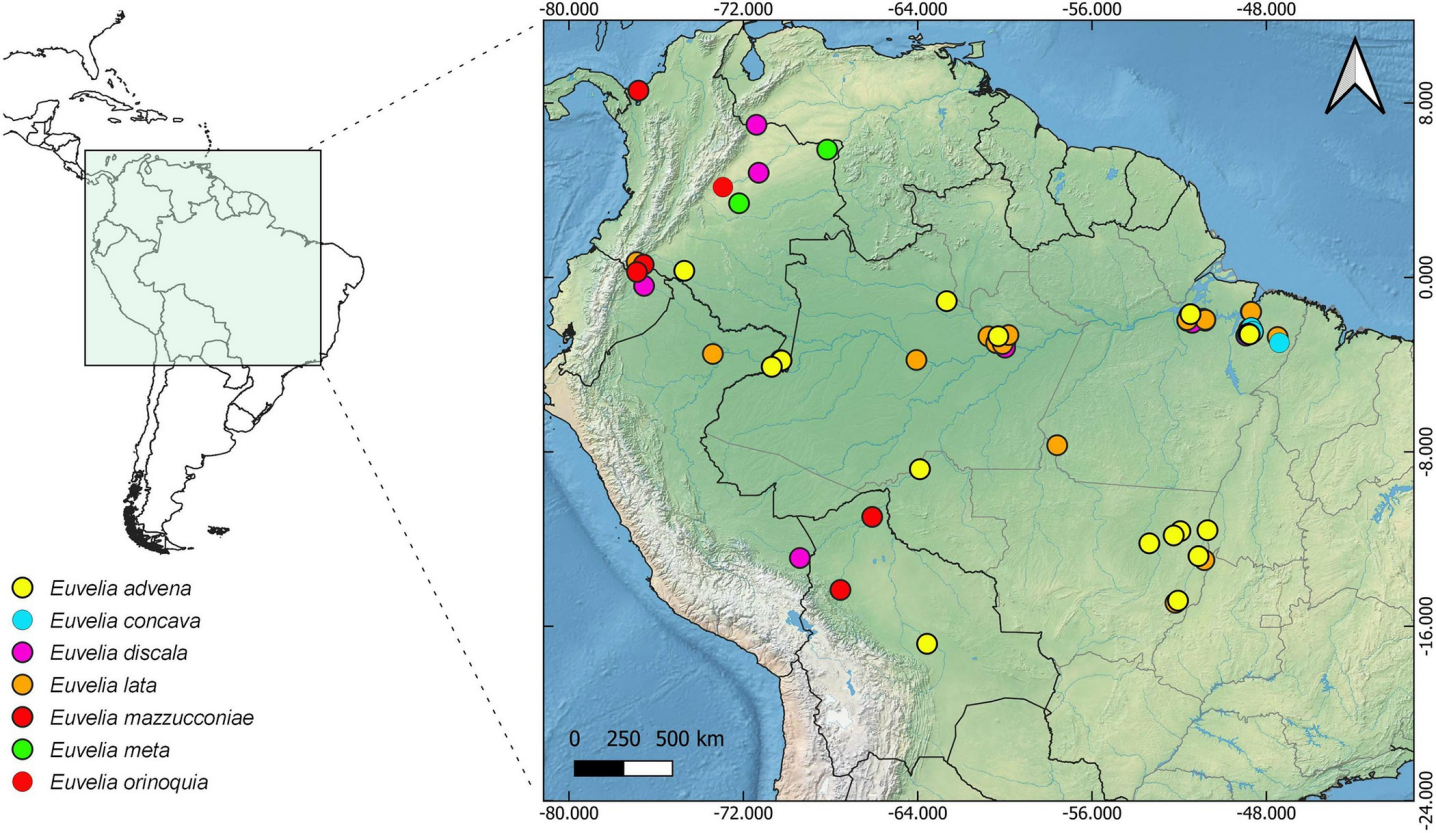
Geographic distribution of *Euvelia*. Spatial data from Natural Earth (http://www.naturalearthdata.com/).

(Figs 7A, 7C, 8 and 9).

#### Type material examined

*Euvelia advena*, holotype. BOLIVIA–[**Santa Cruz**] • Cuatro Ojos, Río Piray; [−16.81, −63.57]; 25.IV.1957; apterous ♂, NMNH. *Euvelia concava*, paratype. BRAZIL–**Mato Grosso** • [Gaúcha do Norte], Capitão Vasconcelos, upper Xingu basin, on River Tuatuari; [−12.201, −53.374]; 31.VII.1957; B. Malkin leg.; 1 apterous ♂, NMNH. *Euvelia discala*, paratype. BRAZIL–**Amazonas** • [Manaus], Ig. [Igarapé] da Arara; [−03.01, −60.40]; 22.XI.1961; E.J. Fittkau leg.; apterous ♂, NMNH. *Euvelia lata*, paratype. BRAZIL–[**Amazonas**] • [Coari / Tefé], Lago Catuá; [−03.79, −64.06]; 12.IX.1961; E.J. Fittkau leg.; apterous ♂, NMNH.

#### Additional material examined

*Euvelia advena*. BRAZIL–**Rondônia** • Porto Velho, Ig. [Igarapé] Bate-Estaca; [−08.81, −63.92]; 22.V.1984; M. Zanuto leg.; 8 apterous ♂, 17 apterous ♀, MPEG. *Euvelia concava*. BRAZIL–**Pará** • Abaetetuba, Ig. [Igarapé] Tijucaquara; [−01.75, −48.84]; 09.VIII.1988; S. Bahia leg.; 1 apterous ♂, 4 apterous ♀, MPEG. *Euvelia mazzucconiae*. BOLIVIA–**El Beni** • Vaca Diéz, Rio Beni [–10.988, –66.089]; X.1937; 19 apterous ♂, 27 apterous ♀, NMNH.

#### Diagnosis

Body length 1.20–2.70. Winged form absent. Teeth between head and thorax (Fig 7C). Male grasping comb short but projected beyond tibial apex. Middle femur 1.3–1.9 times longer than hind femur. Middle tibia 1.3–1.5 times longer than hind tibia. Middle tarsomere I 2.5–4.5 times longer than hind tarsomere I. Middle tarsomere II 1.1–2.0 times longer than middle tarsomere I. Middle pretarsal claws blade-like; middle ventral arolium bipartite into two blade-like structures.

#### Geographic distribution

Colombia, Brazil, Ecuador, Peru, and Bolivia (Fig 9).

#### Comments

According to Andersen [1], the dorsal arolium is usually setiform in Gerromorpha, but may be horizontally flattened like the ventral arolium, which is commonly the longer. The original description of *E*. *mazzucconiae* Aristizábal, Floriano, Moreira & Bispo, 2015 does not mention anything about its arolia (see [5]). Scanning electron micrographs of Bolivian material revealed that the hind ventral arolium is almost as wide as the corresponding claws and that the dorsal arolium is narrower than the ventral but is not setiform (Fig 8D). These modifications have not been detected in other *Euvelia* species studied. This fact, allied to other disparities between *E*. *mazzucconiae* and *E*. *orinoquia* Molano, Moreira & Morales, 2016 (see [5, 6]) and their congeners indicate that species currently held in *Euvelia* might actually belong to two distinct genera

We studied the male terminalia of *E*. *advena* and *E*. *concava* in detail. In both, the posterodorsal margin of abdominal segment VIII bears a weak central notch, while the posteroventral margin is rounded; the proctiger lacks basolateral processes and has the posterior margin rounded; the paramere is short, subequal in length to the proctiger height, with rounded apex; and the phallus has a dorsal and a ventral sclerite. In *E*. *orinoquia*, on the other hand, the paramere is long, as long as the pygophore [6]. This difference in paramere length is one more distinction between the last species and other *Euvelia*. The paramere of *E*. *mazzucconiae* was not mentioned in the original description and was not available for this study. It should be investigated in the future, as well as other aspects of the male terminalia.

### *Husseyella* Herring, 1955

**Fig 10:**
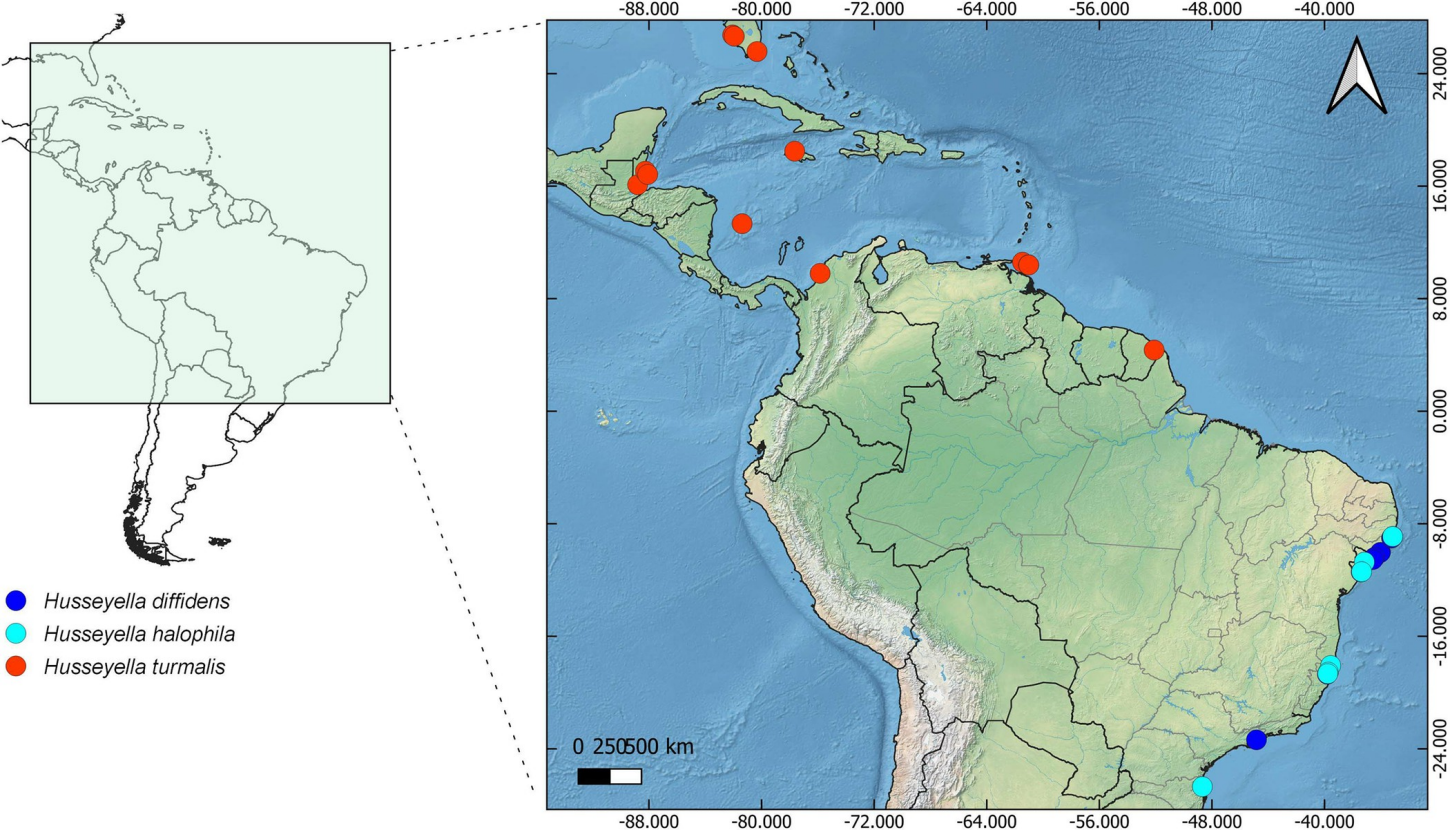
Geographic distribution of *Husseyella*. Spatial from Natural Earth (http://www.naturalearthdata.com/).

(Figs 7B, 7D and 10).

#### Type material examined

*Husseyella diffidens*, paratype. BRAZIL–[**Espírito Santo**] • São Mateus; [−18.7, −39.8]; apterous ♂, NMNH. *Husseyella halophila*, holotype and paratype. BRAZIL–**Santa Catarina** • mouth of small river emptying into the Atlantic; III.1958; F. Plaumann leg.; apterous ♂, apterous ♀, NMNH. *Husseyella turmalis*, paratype. BRITISH HONDURAS [BELIZE]–[**Toledo**] • Punta Gorda [16.10, −88.81]; J.J. White leg.; apterous ♂, NMNH.

#### Additional material examined

*Husseyella diffidens*. BRAZIL–**Pernambuco** • Ilha de Itamaracá, manguezal; −07.6944, −34.8430; 09.III.2018; F.F.F. Moreira, A. Khila & R. Arbore leg.; apterous ♂, CEIOC 81312. *Husseyella halophila*. BRAZIL–**Santa Catarina** • [Barra Velha], Itajubá; [−26.69, −48.69]; IV.1958; F. Plaumann leg.; apterous ♀, MZUSP.–**Alagoas** • Maragogi, Área de Proteção Ambiental Costa dos Corais; −8.9138, −35.1549; 29.IV.2018; C.F.B. Floriano, J.M.S. Rodrigues & O.M. Magalhães leg.; apterous ♂, CEIOC 79735.

#### Diagnosis

Body length 1.99–2.32. Winged form absent. Without teeth between head and thorax (Fig 7C). Male grasping comb short but projected beyond tibial apex. Fore tarsus laterally compressed. Middle femur 1.5 times longer than hind femur. Middle tibia 1.4 times longer than hind tibia. Middle tarsomere I 4.5 times longer than hind tarsomere I. Middle tarsomere II with 70% of middle tarsomere I length. Middle pretarsal claws blade-like; middle ventral arolium bipartite into two blade-like structures.

#### Geographic distribution

Coastal areas of the United States (southern Florida), Mexico, Belize, Jamaica, Trinidad & Tobago, Colombia, French Guiana, and Brazil.

#### Comments

The differences between *Husseyella* and *Euvelia* are subtle, and Armisén *et al*. [3] recovered the three species of the former nested within the latter. Therefore, the two genera might actually be synonyms, with the species currently held in *Husseyella* being just a halophilous lineage within a larger monophyletic clade also containing those currently in *Euvelia*. It is a similar situation as with the now invalid *Trochopus* Carpenter, 1898, which is just a halophilous lineage of *Rhagovelia* Mayr, 1865 (Veliidae: Rhagoveliinae).

Finally, the type specimens of *H*. *halophila* were collected in an unknown coastal water body in the state of Santa Catarina by Fritz Plaumann in March 1958. In the MZUSP, we found a specimen obtained by the same collector in Itajubá Beach, state of Santa Catarina, in April 1958. The type locality of this species might be in the same locality or in a nearby area.
