# Correction: *Xiphovelopsis*, a new South American genus of Microveliinae (Hemiptera, Heteroptera, Gerridae), with the description of a new species

**DOI:** 10.1371/journal.pone.0345121

**Published:** 2026-03-16

**Authors:** Carla Fernanda Burguez Floriano, Juliana Mourão dos Santos Rodrigues, Felipe Ferraz Figueiredo Moreira

The Nomenclatural Acts subsection is missing from the Materials and methods section. The subsection should read as follows: The electronic edition of this article conforms to the requirements of the amended International Code of Zoological Nomenclature, and hence the new names contained herein are available under that Code from the electronic edition of this article. This published work and the nomenclatural acts it contains have been registered in ZooBank, the online registration system for the ICZN. The ZooBank LSIDs (Life Science Identifiers) can be resolved and the associated information viewed through any standard web browser by appending the LSID to the prefix “http://zoobank.org/”. The LSID for this publication is: urn:lsid:zoobank.org:pub:959A2E58-3AD2-46D5-9375-BB2983855EF1. The electronic edition of this work was published in a journal with an ISSN, and has been archived and is available from the following digital repositories: LOCKSS [http://www.lockss.org]; PubMed Central [http://www.ncbi.nlm. nih.gov/pmc].

In the Results and discussion section, the LSID for the new genus and the new species are missing. To ensure their availability, the LSID, names of the new taxa, type designations, and diagnoses are re-declared in this correction. Although without an LSID, the new combination proposed is also re-declared. For detailed descriptions, distributions, etymologies, illustrations, and comments, refer to [1].

***Xiphovelopsis* Floriano & Moreira, gen. nov.**

urn:lsid:zoobank.org:act:42E4F1AB-CA0C-4F80-88F4-D76DDD04DA69

**Diagnosis.** Body length 2.09–2.40. Forewing with four closed cells. Fore femur with long setae ventrally; setae longer than femur width. Male fore tibia with short grasping comb and apical spur. Middle femur with 90–100% hind femur length. Middle tibia with 70–80% hind tibia length. Middle tarsomere I 1.2–1.4 times longer than hind tarsomere I. Middle tarsomere II 1.6–1.9 times longer than middle tarsomere I. Middle pretarsal claws blade-like; middle ventral arolium modified into one blade-like structure. Male paramere long, subequal in length to pygophore height, slightly curved; lateral margins converging; apex acute.

**Type species.** *Microvelia lacunana* Drake & Plaumann, 1953, by present designation.

***Xiphovelopsis tarumana* Floriano & Moreira, sp. nov.**

urn:lsid:zoobank.org:act:5167CED7-01CA-4F62-8BAF-96077803C66F

**Diagnosis.** Body length 2.40; body color mainly brown; venter of male abdominal segment VIII with a pair of depressions and a row of setae.

**Type material examined.** Holotype. BRAZIL–**Amazonas** • Manaus, Tarumã-Mirim, Rio Negro; [−02.97, −60.20]; 03-05.X.2002; D.L.V. Pereira leg.; apterous ♂, Coleção de Invertebrados, Instituto Nacional de Pesquisas da Amazônia, Manaus, Brazil (INPA).

***Xiphovelopsis lacunana* (Drake & Plaumann, 1953) comb. nov.**

**Diagnosis.** Body length 2.09; body color mainly black; abdominal sternum VII covered by golden setae; venter of male abdominal segment VIII with one depression, without row of setae.
